# Supplementary material for: Targeting GLP-1 receptor trafficking to improve agonist efficacy
Source: Nat Commun. 2018 Apr 23;9:1602. doi: 10.1038/s41467-018-03941-2 (PMC5913239; doi:10.1038/s41467-018-03941-2)
Supplement: Supplementary file 1 — Supplementary Information [file 41467_2018_3941_MOESM1_ESM.pdf]

Targeting GLP-1 receptor trafficking to improve agonist efficacy. Jones et al.

a

Ex4	H	G	E	G	T	F	T	S	D	L	S	K	Q	M	E	E	E	A	V	R	L	F	I	E	W	L	K	N	G	G	P	S	S	G	A	P	P	P	S	NH2
ex-dHis1	**dH**	G	E	G	T	F	T	S	D	L	S	K	Q	M	E	E	E	A	V	R	L	F	I	E	W	L	K	N	G	G	P	S	S	G	A	P	P	P	S	NH2
ex-asn1	**N**	G	**E**	G	T	F	T	S	D	L	S	K	Q	M	E	E	E	A	V	R	L	F	I	E	W	L	K	N	G	G	P	S	S	G	A	P	P	P	S	NH2
ex-gln1	**Q**	G	E	G	T	F	T	S	D	L	S	K	Q	M	E	E	E	A	V	R	L	F	I	E	W	L	K	N	G	G	P	S	S	G	A	P	P	P	S	NH2
ex-tyr1	**Y**	G	E	G	T	F	T	S	D	L	S	K	Q	M	E	E	E	A	V	R	L	F	I	E	W	L	K	N	G	G	P	S	S	G	A	P	P	P	S	NH2
ex-dTyr1	**dY**	G	E	G	T	F	T	S	D	L	S	K	Q	M	E	E	E	A	V	R	L	F	I	E	W	L	K	N	G	G	P	S	S	G	A	P	P	P	S	NH2
ex-phe1	**F**	G	E	G	T	F	T	S	D	L	S	K	Q	M	E	E	E	A	V	R	L	F	I	E	W	L	K	N	G	G	P	S	S	G	A	P	P	P	S	NH2
ex-ala2	H	**A**	E	G	T	F	T	S	D	L	S	K	Q	M	E	E	E	A	V	R	L	F	I	E	W	L	K	N	G	G	P	S	S	G	A	P	P	P	S	NH2
ex-AIB2	H	**AIB**	E	G	T	F	T	S	D	L	S	K	Q	M	E	E	E	A	V	R	L	F	I	E	W	L	K	N	G	G	P	S	S	G	A	P	P	P	S	NH2
ex-asp3	H	G	**D**	G	T	F	T	S	D	L	S	K	Q	M	E	E	E	A	V	R	L	F	I	E	W	L	K	N	G	G	P	S	S	G	A	P	P	P	S	NH2
ex-gln3	H	G	**Q**	G	T	F	T	S	D	L	S	K	Q	M	E	E	E	A	V	R	L	F	I	E	W	L	K	N	G	G	P	S	S	G	A	P	P	P	S	NH2
ex-dGln3	H	G	**dQ**	G	T	F	T	S	D	L	S	K	Q	M	E	E	E	A	V	R	L	F	I	E	W	L	K	N	G	G	P	S	S	G	A	P	P	P	S	NH2

Exendin(9-39)	D	L	S	K	Q	M	E	E	E	A	V	R	L	F	I	E	W	L	K	N	G	G	P	S	S	G	A	P	P	P	S	NH2
GLP-1(7-36)NH2	H	A	**E**	G	T	F	T	S	D	V	S	S	Y	L	E	G	Q	A	A	K	E	F	I	A	W	L	V	K	G	R	NH2	

b

	Internalization CHO-SNAP-GLP-1R			cAMP PathHunter CHO-GLP-1R			β-arrestin-2 PathHunter CHO-GLP-1R		
	$LogEC_{50}$ (M)	E_{max} (% loss)	Hill slope	$LogEC_{50}$ (M)	E_{max} (% GLP-1)	Hill slope	$LogEC_{50}$ (M)	E_{max} (% GLP-1)	Hill slope
ex4	-8.2 (0.1)	83 (7)	2.1 (0.3)	-10.3 (0.0)	105 (3)	1.7 (0.1)	-7.7 (0.1)	97 (8)	1.0 (0.1)
ex-dHis1	-7.6* (0)	73** (3)	1.2 (0.2)	-10.3 (0.0)	96 (3)	1.9 (0.3)	-7.1 (0.1)	48*** (5)	0.9 (0.1)
ex-asn1	-7.2** (0.3)	41*** (4)	5.0 (4)	-9.8 *** (0.1)	101 (4)	1.6 (0.1)	-7.1 (0.1)	15*** (2)	1.5 (0.2)
ex-gln1	N.C.	22*** (5)	N.C.	-9.2*** (0.0)	105 (2)	2 (0.1)	-6.4*** (0.3)	6*** (1)	1.4 (0.4)
ex-tyr1	-7.7* (0.3)	42*** (5)	1.9 (0.6)	-9.7*** (0.0)	96 (4)	2.1 (0.1)	-7.0* (0.2)	12*** (1)	1.1 (0.2)
ex-dTyr1	N.C.	7*** (6)	N.C.	-9.2*** (0.1)	99 (6)	2.1 (0.4)	-6.3*** (0.3)	5*** (1)	1.2 (0.5)
ex-phe1	-7.2*** (0)	28** (9)	2.1 (1.3)	-9.9*** (0.1)	99 (3)	2.4 (0.6)	-7.3 (0.1)	12*** (1)	1.2 (0.1)
ex-ala2	-8.2 (0.1)	88* (3)	1.8 (0.2)	-9.8*** (0.0)	106 (3)	1.9 (0.3)	-7.6 (0.0)	99 (9)	1.3 (0.2)
ex-AIB2	-8.4 (0)	82 (2)	3.2 (1.7)	-9.8*** (0.1)	104 (4)	1.8 (0.3)	-7.6 (0.0)	89 (5)	1.4 (0.2)
ex-asp3	-8.2 (0.1)	91** (3)	1.5 (0.2)	-9.9*** (0.0)	110 (12)	1.4 (0.2)	-7.7 (0.1)	98 (9)	1.1 (0.1)
ex-gln3	-8.4 (0.1)	84 (3)	1.5 (0.2)	-10.2 (0.1)	113 (18)	1.6 (0.4)	-7.8 (0.1)	81 (10)	1.4 (0.2)
ex-dGln3	-7.4*** (0.1)	70 (7)	3.1 (1.8)	-10.0*** (0.1)	97 (3)	2.7 (0.9)	-7.1* (0.1)	31*** (4)	1.1 (0.2)

c

	16 h insulin secretion				1 h insulin secretion			
	INS-1 832/3			MIN6B1	INS-1 832/3			MIN6B1
	LogEC$_{50}$ (M)	E$_{max}$ (ISI)	Hill slope	ISI	LogEC$_{50}$ (M)	E$_{max}$ (ISI)	Hill slope	ISI
ex4	-11.4 (0.3)	2.8 (0.4)	0.64 (0.05)	1.8 (0.2)	-9.9 (0.4)	1.9 (0.1)	0.66 (0.03)	1.5 (0.1)
ex-dHis1	-10.0** (0.3)	4.0** (0.6)	shared	2.0 (0.2)	-10.8 (0.4)	1.6 (0.1)	shared	1.4 (0.1)
ex-asn1	-8.5*** (0.4)	4.8*** (0.7)	shared	2.1 (0.2)	-10.2 (0.5)	1.6 (0.1)	shared	1.4 (0.1)
ex-gln1	-8.2*** (0.2)	4.3*** (0.6)	shared	2.1 (0.2)	-8.4 (0.6)	1.9 (0.4)	shared	1.4 (0.1)
ex-tyr1	-9.5*** (0.2)	4.0** (0.6)	shared	2.1** (0.2)	-9.8 (0.6)	1.5 (0.1)	shared	1.4 (0.1)
ex-dTyr1	-8.8*** (0.3)	4.3*** (0.6)	shared	2.1** (0.2)	-9.0 (0.7)	2.0 (0.3)	shared	1.4 (0.1)
ex-phe1	-9.6*** (0.3)	4.4*** (0.6)	shared	2.1** (0.2)	-8.6 (0.4)	2.0 (0.2)	shared	1.5 (0.0)
ex-ala2	-10.7 (0.7)	1.6** (0.2)	shared	1.5*** (0.1)	-9.9 (0.4)	1.7 (0.1)	shared	1.4 (0.1)
ex-AIB2	-11.1 (0.5)	2.4 (0.4)	shared	1.9 (0.2)	-9.7 (0.4)	2.0 (0.2)	shared	1.4 (0.0)
ex-asp3	-10.7 (0.6)	1.7* (0.2)	shared	1.5*** (0.2)	-9.2 (0.5)	1.7 (0.2)	shared	1.4 (0.0)
ex-gln3	-11.0 (0.4)	2.6 (0.4)	shared	1.8 (0.2)	-9.8 (0.4)	1.8 (0.2)	shared	1.4 (0.1)
ex-dGln3	-9.6*** (0.2)	4.0** (0.6)	shared	2.0 (0.2)	-9.3 (0.7)	1.9 (0.2)	shared	1.5 (0.1)

Supplementary Figure 1: Agonist sequences and screen results.

(**a**) Agonist sequences using single letter amino acid code, including parent molecule exendin-4 ("ex4"). (**b**) Agonist pharmacological characteristics, including GLP-1R internalization in CHO-SNAP-GLP-1R cells (90 min, E_{max} expressed as % surface receptor loss, *n*=5), cAMP potency in PathHunter CHO-GLP-1R cells (90 min, E_{max} expressed relative to GLP-1, *n*=5), β-arrestin-2 recruitment in PathHunter CHO-GLP-1R cells (90 min, E_{max} expressed relative to GLP-1, *n*=5). Curve fitting parameter estimates from 4-parameter logistic fit, compared by one-way randomized block ANOVA with Dunnett's test *vs.* exendin-4. (**c**) Prolonged (16 h) and acute (1 h) agonist insulin secretion results in INS-1 832/3 and MIN6B1 cells, E_{max} expressed as insulin secretion index ("ISI"), i.e. fold increase *vs.* 11 mM glucose alone, *n*=5-7. Parameter estimates from 4-parameter logistic fit, with Hill slope globally constrained for each assay, compared by one-way randomized block ANOVA with Dunnett's test *vs.* exendin-4. * p<0.05, ** p<0.01, *** p<0.001 *vs.*

exendin-4 using statistical test indicated above. SEM indicated in brackets. N.C. = not calculable (in case of very low efficacy agonists).

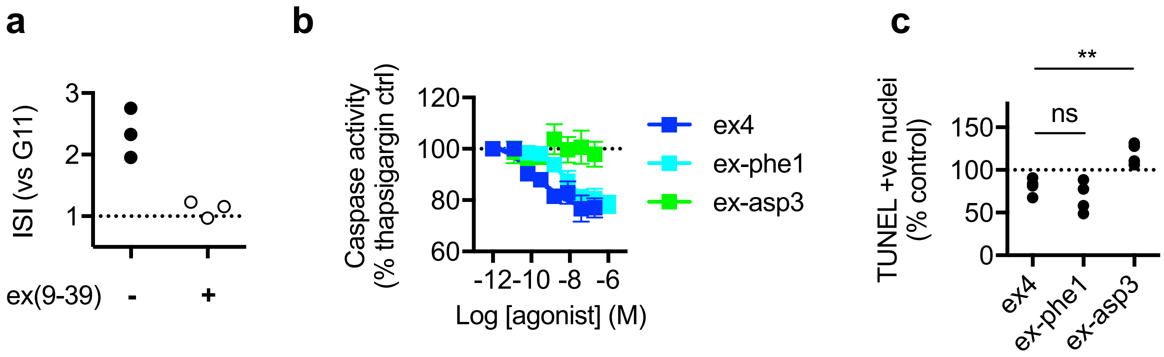

Supplementary Figure 2: Additional beta cell testing.

(**a**) Insulin release during overnight incubation with exendin-phe1 ± 10 µM exendin(9-39), *n*=3. (**b**) Dose response for apoptosis inhibition in INS-1 832/3 cells treated overnight with 1 µM thapsigargin to induce ER stress, expressed relative to thapsigargin alone, *n*=6, 4-parameter logistic fit of averaged data shown. (**c**) Effect of test agonists on apoptosis induced by glucolipotoxicity (25 mM glucose, 0.5 mM BSA-palmitate overnight) in MIN6B1 cells, as determined by TUNEL assay, expressed relative to high glucose/palmitate alone, *n*=4, one-way ANOVA with Dunnett's test *vs.* exendin-4. Agonists applied at 100 nM unless indicated. ** p<0.01 *vs.* exendin-4 using statistical test indicated above. Error bars indicate SEM.

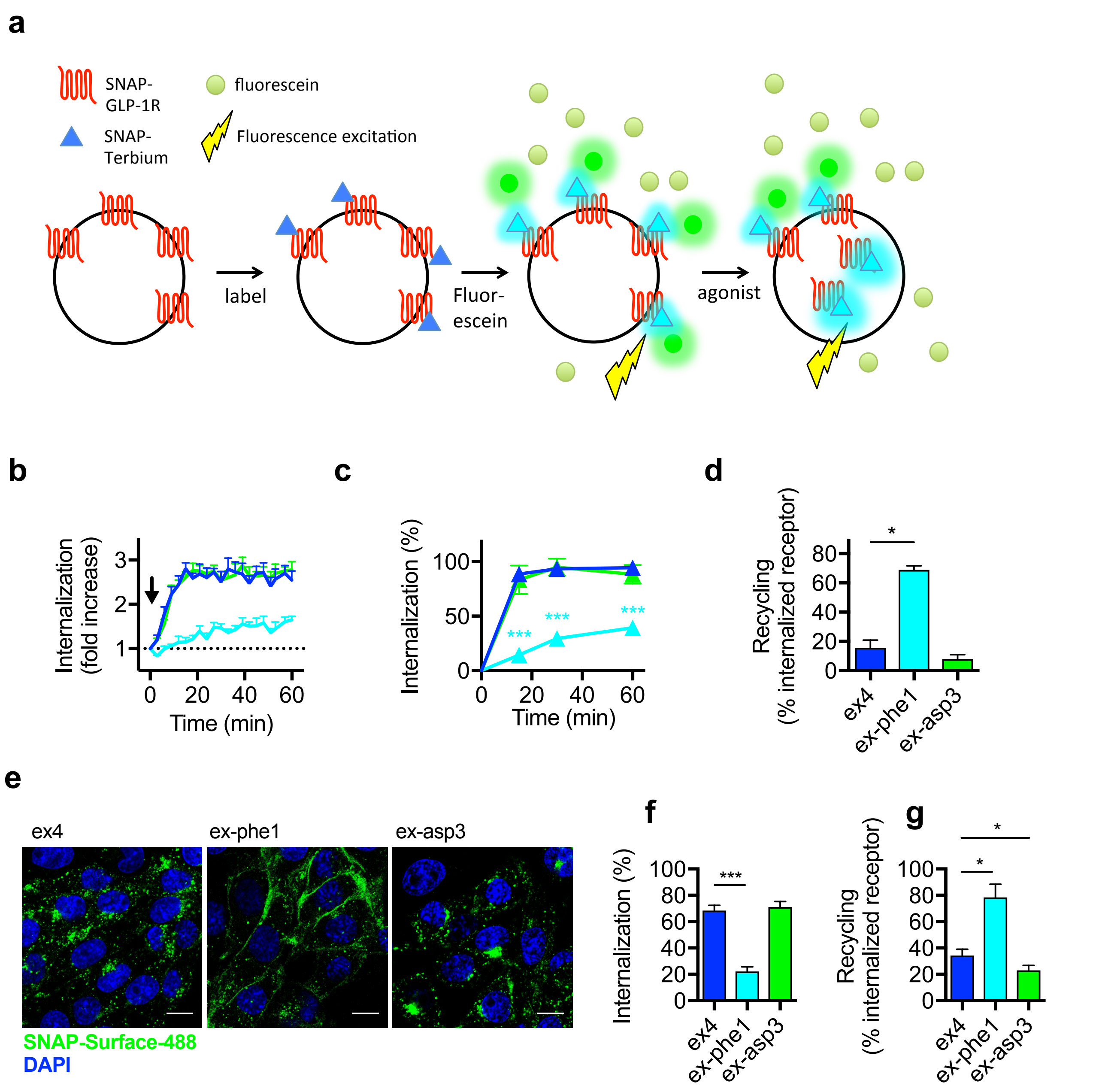

Supplementary Figure 3: GLP-1R trafficking in CHO-SNAP-GLP-1R cells.

(**a**) Schematic describing DERET assay. (**b**) Agonist-induced SNAP-GLP-1R internalization in CHO-SNAP-GLP-1R cells quantified by diffusion-enhanced resonance energy transfer (DERET) assay, indicated as fold signal increase from baseline, *n*=6. (**c**) Internalization in CHO-SNAP-GLP-1R cells, quantified by FACS as in Fig. 2b, *n*=3, two-way ANOVA with Dunnett's test *vs.* exendin-4**.** (**d**) Recycling (30 min) after an initial 15 min agonist exposure in CHO-SNAP-GLP-1R cells, quantified by FACS as above, *n*=3, one-way ANOVA with Dunnett's test *vs.* exendin-4. (**e**) Representative images of CHO-SNAP-GLP-1R cells, labeled with SNAP-Surface 488 and treated with agonist for 30 min, to induce internalization, *n*=2; scale bars, 8 µm. (**f**) Agonist-induced GLP-

1R internalization measured by post-stimulation surface labeling with Lumi4-Tb in a plate reader, 60 min stimulation, *n*=5, one-way randomized block ANOVA with Dunnett's test *vs.* exendin-4. (**g**) As for (f) but with further 60 min recycling in the presence of 10 μM exendin(9-39), *n*=6. Agonists applied at 100 nM. * $p<0.05$, *** $p<0.001$ *vs*. exendin-4 using statistical test indicated above. Error bars indicate SEM.

a

ex4	H	G	E	G	T	F	T	S	D	L	S	K-FITC	Q	M	E	E	E	A	V	R	L	F	I	E	W	L	K	N	G	G	P	S	S	G	A	P	P	P	S	NH2
ex-phe1	F	G	E	G	T	F	T	S	D	L	S	K-FITC	Q	M	E	E	E	A	V	R	L	F	I	E	W	L	K	N	G	G	P	S	S	G	A	P	P	P	S	NH2
ex-asp3	H	G	D	G	T	F	T	S	D	L	S	K-FITC	Q	M	E	E	E	A	V	R	L	F	I	E	W	L	K	N	G	G	P	S	S	G	A	P	P	P	S	NH2

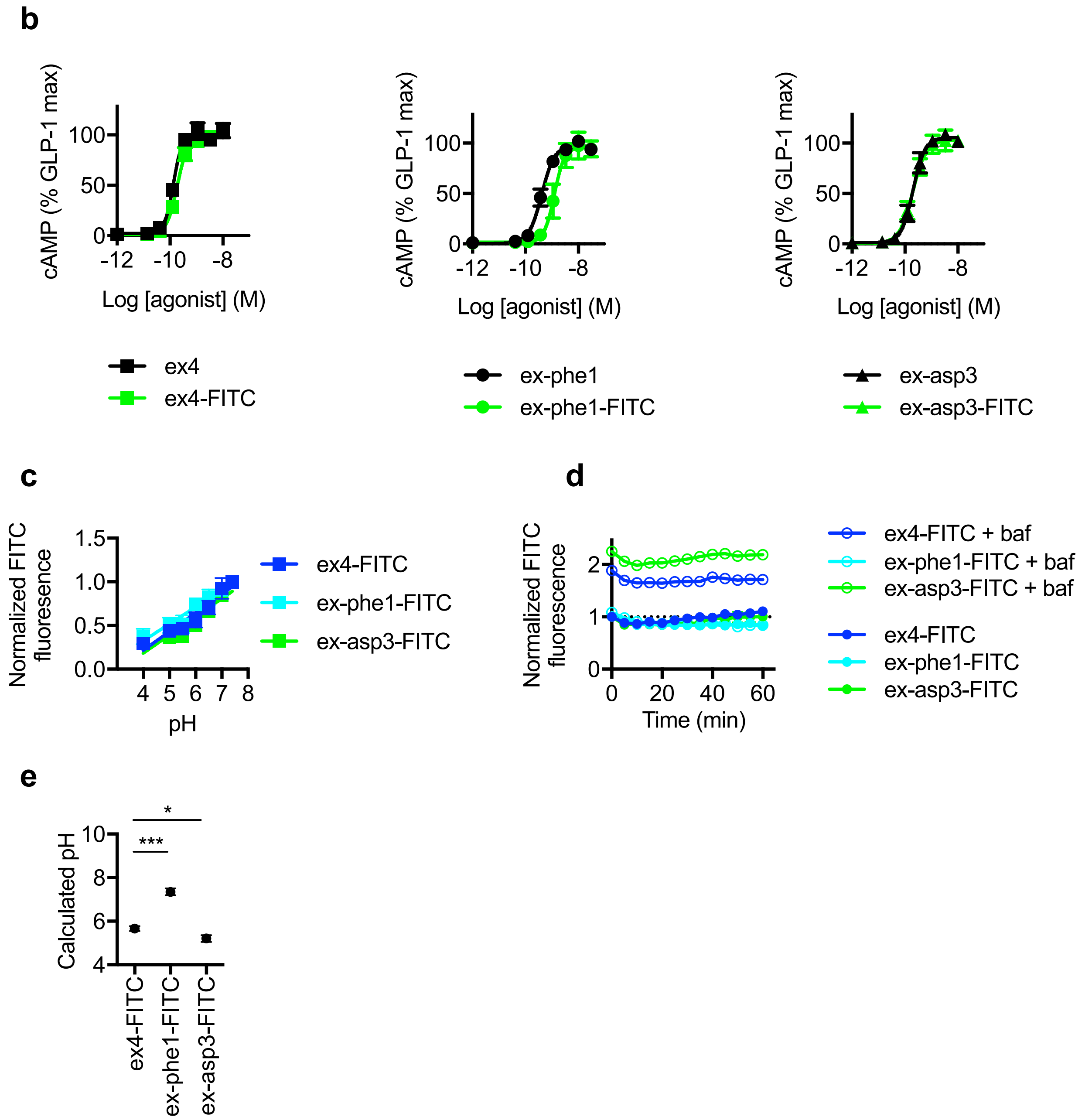

Supplementary Figure 4: FITC-agonists.

(**a**) FITC-agonist sequences denoted by single letter amino acid code. (**b**) cAMP responses in CHO-SNAP-GLP-1R cells demonstrating agonist potency for FITC-conjugated *vs.* unmodified agonists, 30 min incubation, *n*=3. (**c**) Effect of pH on FITC-agonist fluorescence in solution, normalized to pH 7.4 value, data fitted by linear regression, *n*=3. (**d**) Direct measurement of FITC fluorescence from internalized FITC-agonist (30 min, 100 nM) in CHO-SNAP-GLP-1R cells ±

bafilomycin ("baf", 100 nM), measured over 60 min and normalized to baseline reading without bafilomycin, *n*=4. (**e**) Estimated average pH of FITC-agonist after 30 min internalization, by extrapolation of data from (d) to calibration shown in (c), *n*=4. * $p<0.05$, *** $p<0.001$ by one-way randomized block ANOVA with Dunnett's test *vs*. exendin-4. Error bars indicate SEM.

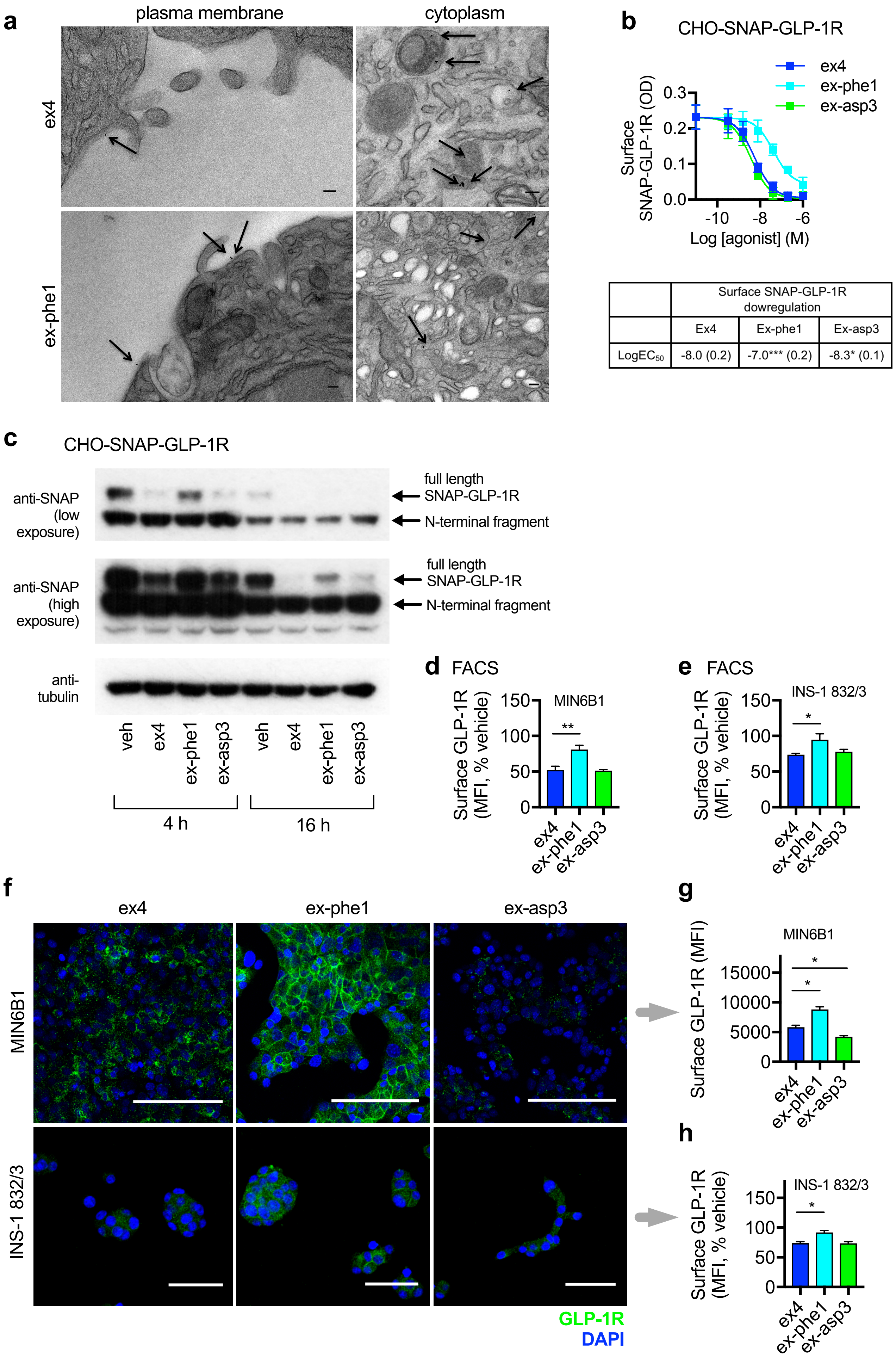

	Surface SNAP-GLP-1R dowregulation		
	Ex4	Ex-phe1	Ex-asp3
$LogEC_{50}$	-8.0 (0.2)	-7.0*** (0.2)	-8.3* (0.1)

Supplementary Figure 5: Further analysis of surface GLP-1R down-regulation and degradation.

(**a**) Representative electron micrographs showing typical subcellular localization of SNAP-GLP-1R (labeled with cleavable SNAP-Surface-biotin plus streptavidin-10 nm-gold, arrows), 60 min agonist exposure; scale bars, 0.1 µm; images are from same experiments as in Fig. 2f. (**b**) Surface down-regulation of GLP-1R measured by surface ELISA in CHO-GLP-1R cells exposed to agonist for 16 h, *n*=5, table indicates relative agonist potencies compared by one-way randomized block ANOVA with Dunnett's test *vs.* exendin-4. (**c**) Immunoblot indicating agonist-induced degradation of GLP-1R in CHO-SNAP-GLP-1R cells after 4 and 16 h agonist exposure, representative result from *n*=2 experiments. (**d**) Surface down-regulation of GLP-1R in MIN6B1 cells, residual surface receptor labeled after 16 h agonist treatment and quantified by FACS, results normalized to vehicle control, *n*=4, one-way ANOVA with Dunnett's test *vs.* exendin-4. (**e**) As for (d), but for INS-1 832/3 cells, *n*=4. (**f**) Immunofluorescence images demonstrating surface GLP-1R down-regulation in MIN6B1 and INS-1 832/3 cells; residual surface receptor labeled after 16 h agonist treatment; scale bars, 100 µm (MIN6B1) and 50 µm (INS-1 832/3). (**g**) and (**h**) Quantification of residual surface GLP-1R in experiments shown in (f), 5 images analyzed from *n*=3-5 coverslips, mean cellular fluorescence indicated (normalized for INS-1 832/3 to exendin-4), one-way ANOVA with Dunnett's test *vs.* exendin-4. Agonists applied at 100 nM except where indicated. * $p<0.05$, *** $p<0.001$, by statistical test indicated above. Error bars indicate SEM.

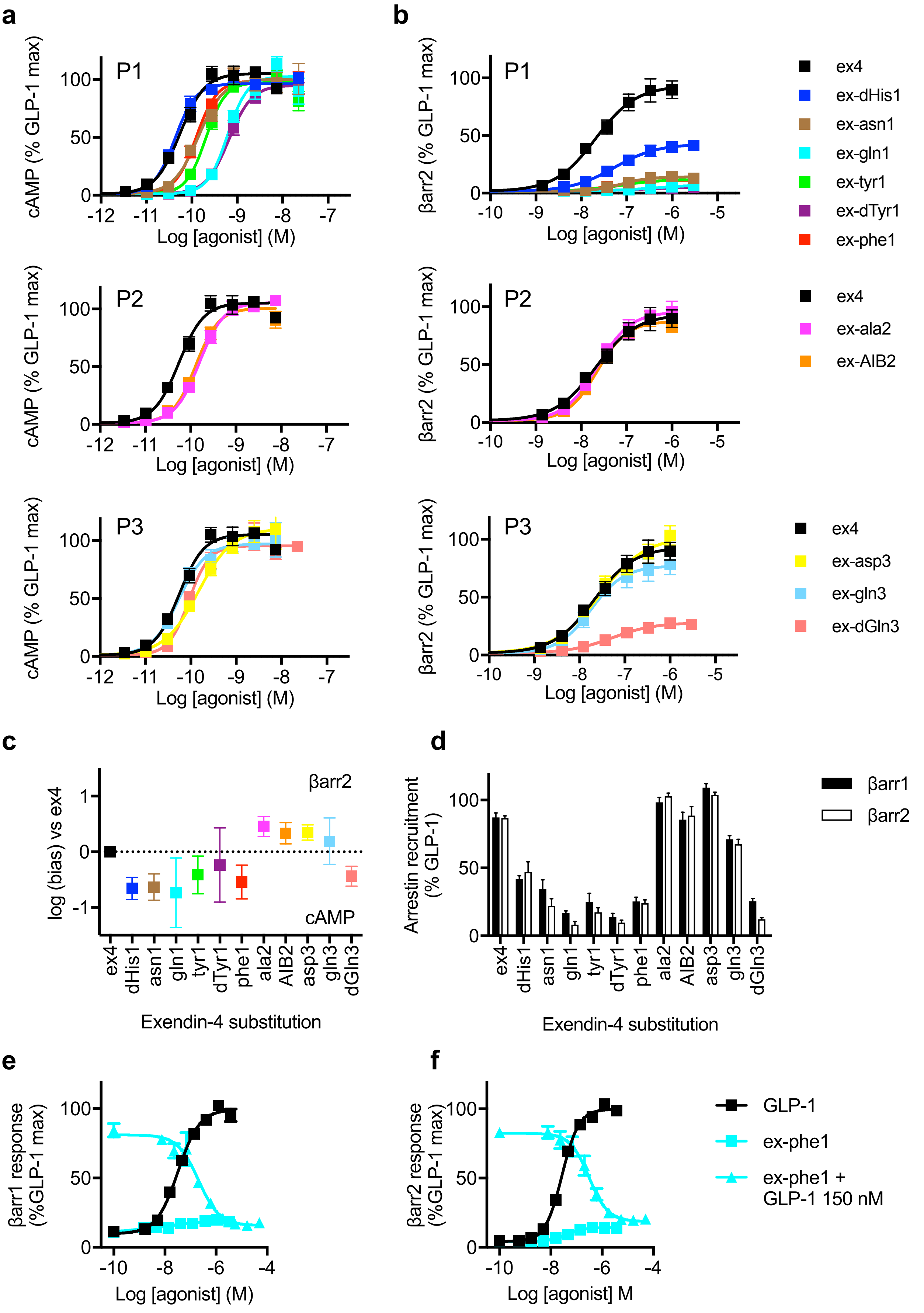
a
b
P1
P2
P3
cAMP (% GLP-1 max)
βarr2 (% GLP-1 max)
Log [agonist] (M)
ex4
ex-dHis1
ex-asn1
ex-gln1
ex-tyr1
ex-dTyr1
ex-phe1
ex-ala2
ex-AIB2
ex-asp3
ex-gln3
ex-dGln3
c
d
log (bias) vs ex4
βarr2
cAMP
Arrestin recruitment (% GLP-1)
βarr1
βarr2
Exendin-4 substitution
ex4
dHis1
asn1
gln1
tyr1
dTyr1
phe1
ala2
AIB2
asp3
gln3
dGln3
e
f
βarr1 response (%GLP-1 max)
βarr2 response (%GLP-1 max)
Log [agonist] (M)
Log [agonist] M
GLP-1
ex-phe1
ex-phe1 + GLP-1 150 nM

Supplementary Figure 6: Biased signaling.

Note – these data are also summarized in Supplementary Fig. 1b. (**a**) cAMP responses, 90 min incubation, *n*=5, graphs arranged by substitution position (position 1 = "P1", etc.). (**b**) As in (a) but for β-arrestin-2 recruitment. (**c**) Signaling bias, quantified as $\Delta\Delta\log(\tau/K_A)$ using data from (a) and (b), error bars indicate 95% CI. (**d**) Comparison of β-arrestin1 and β-arrestin-2 responses, 1 μM agonist, 90 min incubation, expressed relative to GLP-1 response, *n*=3. Competitive antagonism by exendin-phe1 against GLP-1-induced (**e**) β-arrestin-1 recruitment, *n*=5, and (**f**) β-arrestin-2 recruitment, *n*=6. Exendin-phe1 and GLP-1 (150 nM) applied simultaneously for 90 min. All experiments performed in PathHunter CHO-GLP-1R cells. Except where indicated (bias plot), error bars indicate SEM.

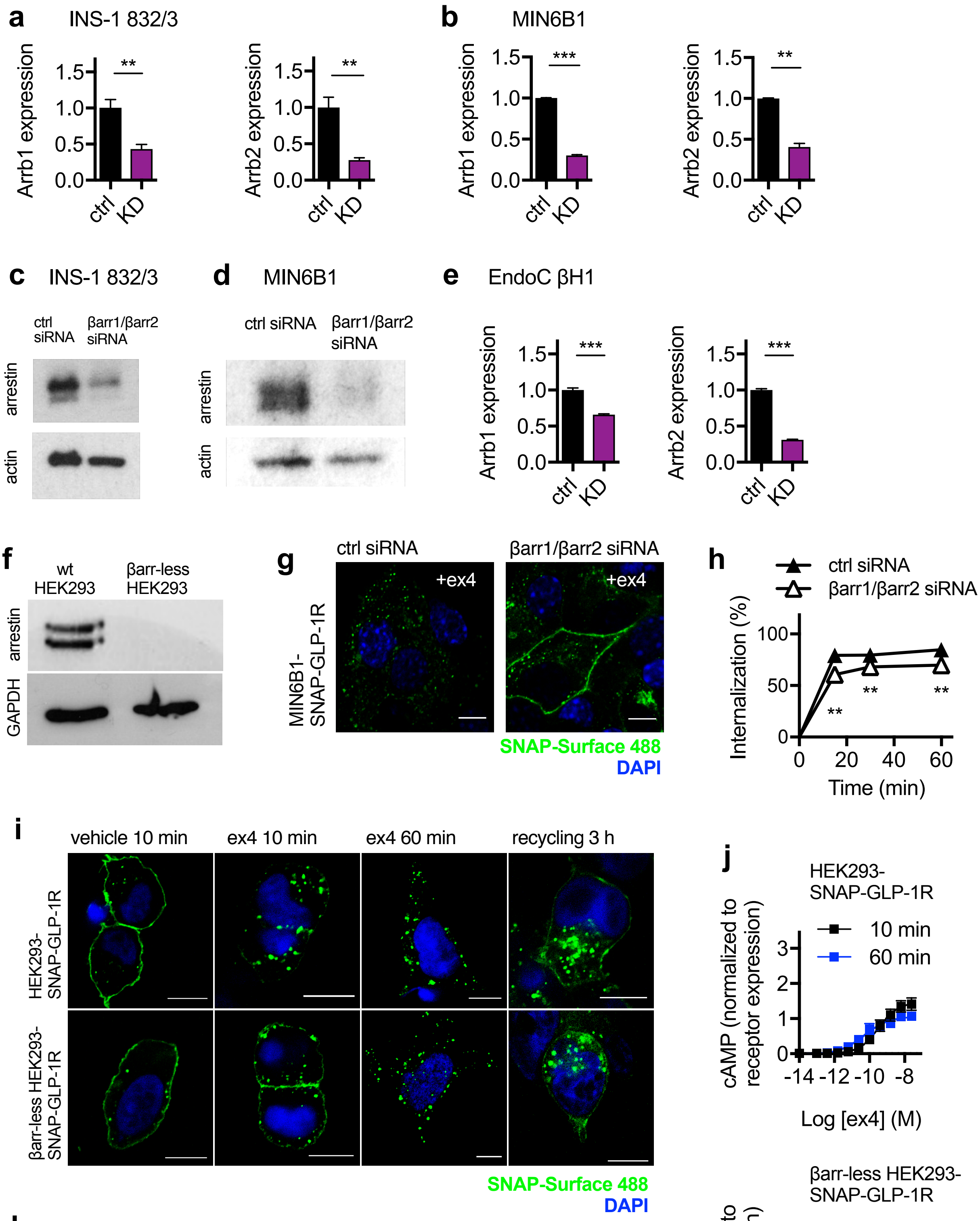

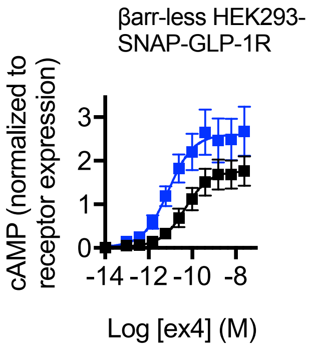

k

	Wildtype HEK293-SNAP-GLP-1R			βarr-less HEK293-SNAP-GLP-1R		
	$LogEC_{50}$	E_{max}	Hill slope	$LogEC_{50}$	E_{max}	Hill slope
10 min	-9.5 (0.13)	1.47 (0.17)	0.83 (0.06)	-10.3* (0.15)	1.78 (0.35)	0.92 (0.15)
60 min	-10.2 (0.27)	1.08 (0.12)	0.97 (0.23)	-11.2* (0.16)	2.60*** (0.52)	0.95 (0.15)

Supplementary Figure 7: Effects of dual β-arrestin silencing.

(**a**) Knockdown efficiency determined by qRT-PCR in INS-1 832/3 cells at 72 h, relative to control (ctrl) RNAi, *n*=4, two-tailed t-test. (**b**) As for (a) but in MIN6B1. (**c**) Western blot demonstrating β-arrestin-1/2 protein knockdown in INS-1 832/3 cells at 72 h; 60.2% knockdown achieved after normalization for loading control. (**d**) As in (c) but for MIN6B1 cells, 77.3% knockdown achieved after normalization for loading control. (**e**) Knockdown efficiency in EndoC-βH1 lentivirally transduced with β-arrestin-1 and β-arrestin-2 shRNA, *n*=3, two-tailed t-test. (**f**) Western blot showing β-arrestin-1/2 knockout in β-arrestin-less ("βarr-less") *vs.* wild-type HEK293 cells. (**g**) Representative image demonstrating delayed SNAP-GLP-1R internalization after dual β-arrestin knockdown in MIN6B1-SNAP-GLP-1R cells labeled with SNAP-Surface 488 prior to 15 min treatment with exendin-4; scale bars, 10 μm, *n*=2. (**h**) Effect of dual β-arrestin silencing on exendin-4-induced GLP-1R internalization (by FACS) in MIN6B1-SNAP-GLP-1R cells, *n*=3, two-way randomized block ANOVA with Sidak's test. (**i**) Delayed SNAP-GLP-1R endocytosis in β-arrestin-less *vs.* wild-type HEK293 cells stably expressing SNAP-GLP-1R, labeled before agonist stimulation with SNAP-Surface-488, *n*=3. (**j**) cAMP responses to exendin-4 in β-arrestin-less *vs.* wild-type HEK293-SNAP-GLP-1R cells at 10 min and 60 min, no IBMX, *n*=3. (**k**) Quantification of responses shown in (i), curve fitting by 4-parameter logistic fit, parameters compared with paired two-way ANOVA with Sidak's test for β-arrestin-less *vs.* wild-type. Agonists applied at 100 nM except where indicated. * $p<0.05$, ** $p<0.01$, *** $p<0.001$ by statistical test defined in the text. Error bars (and bracketed numbers in table) indicate SEM.

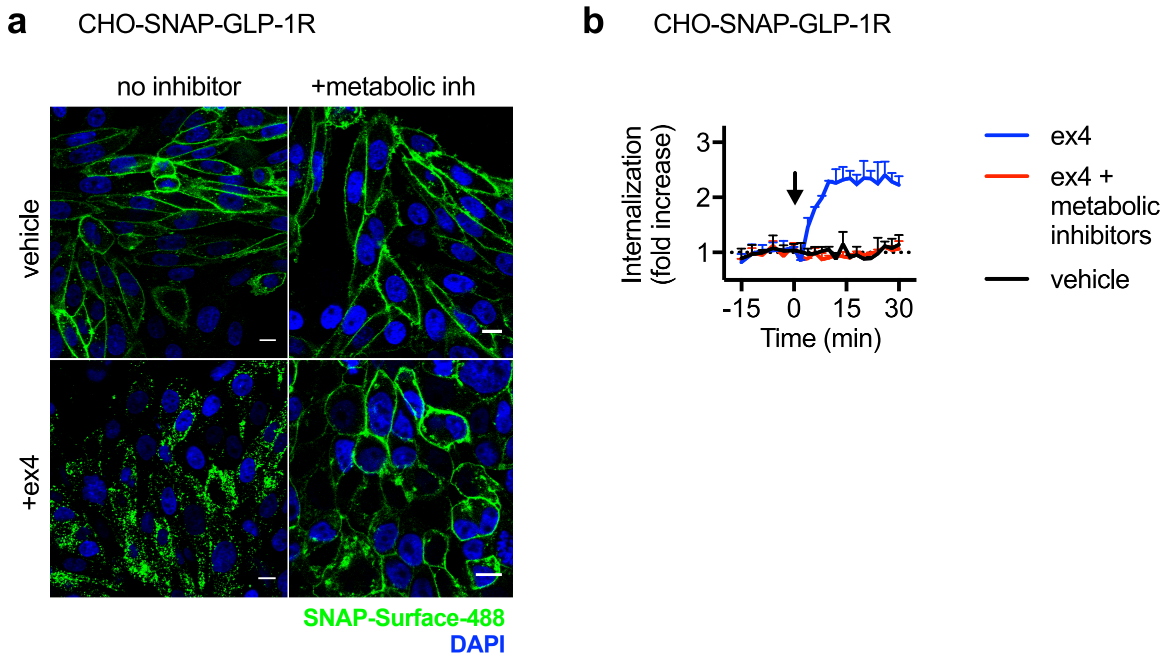

Supplementary Figure 8: Inhibition of endocytosis.

(**a**) Microscopy demonstrating effect of metabolic inhibitor cocktail (20 min pre-treatment) on 100 nM, 10 min exendin-4-induced internalization in CHO-SNAP-GLP-1R cells. (**b**) As for (a) but measurement by DERET, *n*=2. Error bars indicate SEM.

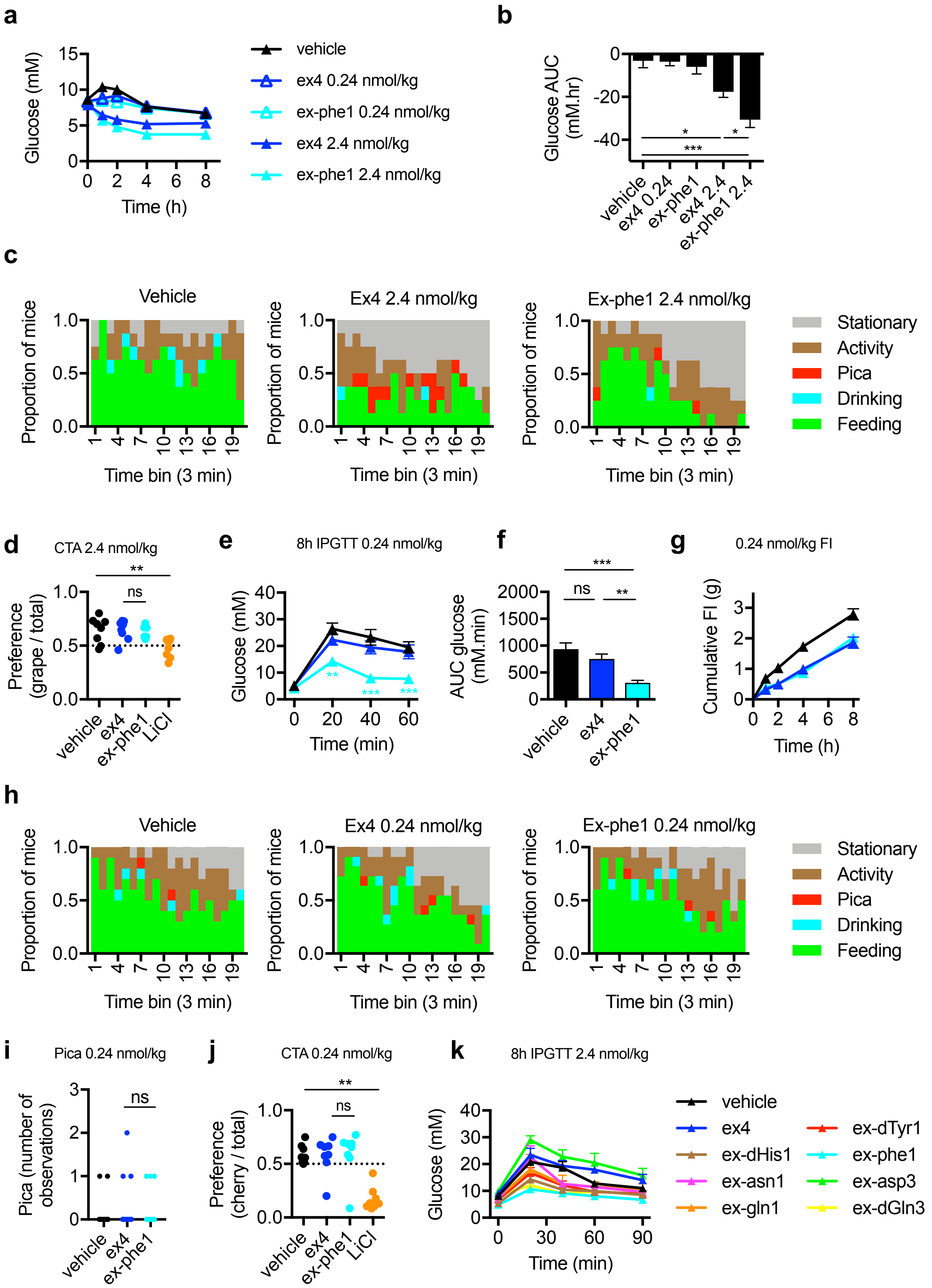

a
Glucose (mM)
Time (h)
vehicle
ex4 0.24 nmol/kg
ex-phe1 0.24 nmol/kg
ex4 2.4 nmol/kg
ex-phe1 2.4 nmol/kg
b
Glucose AUC (mM.hr)
vehicle
ex4 0.24
ex-phe1
ex4 2.4
ex-phe1 2.4
c
Vehicle
Ex4 2.4 nmol/kg
Ex-phe1 2.4 nmol/kg
Proportion of mice
Time bin (3 min)
Stationary
Activity
Pica
Drinking
Feeding
d
CTA 2.4 nmol/kg
Preference (grape / total)
vehicle
ex4
ex-phe1
LiCl
e
8h IPGTT 0.24 nmol/kg
Glucose (mM)
Time (min)
f
AUC glucose (mM.min)
vehicle
ex4
ex-phe1
g
0.24 nmol/kg FI
Cumulative FI (g)
Time (h)
h
Vehicle
Ex4 0.24 nmol/kg
Ex-phe1 0.24 nmol/kg
Proportion of mice
Time bin (3 min)
Stationary
Activity
Pica
Drinking
Feeding
i
Pica 0.24 nmol/kg
Pica (number of observations)
vehicle
ex4
ex-phe1
j
CTA 0.24 nmol/kg
Preference (cherry / total)
vehicle
ex4
ex-phe1
LiCl
k
8h IPGTT 2.4 nmol/kg
Glucose (mM)
Time (min)
vehicle
ex4
ex-dHis1
ex-asn1
ex-gln1
ex-dTyr1
ex-phe1
ex-asp3
ex-dGln3

l

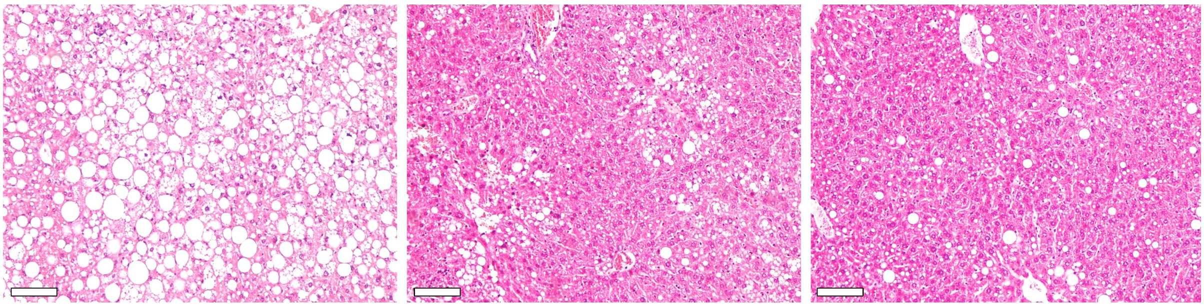

Supplementary Figure 9: Further in vivo results.

(**a**) Effect of single IP agonist injection at the indicated dose on blood glucose in HFHS mice, *n*=9-10 per group. (**b**) AUC calculated from (a), relative to baseline glucose at t=0, one-way ANOVA with Tukey's test. (**c**) Behavioral satiety study in fasted lean mice injected with 2.4 nmol/kg agonist IP, with indicated behaviors monitored after return of food provision, at 3-minute intervals for 60 min, *n*=7-8 per treatment. (**d**) Conditioned taste aversion in lean mice trained to associated indicated stimulus with grape Kool-Aid flavor and then given free choice of water or Kool-Aid; agonist dose 2.4 nmol/kg, LiCl (0.15 M in a volume equivalent to 2% body weight) used as positive control; *n*=8/group, one-way ANOVA with Holm-Sidak test. (**e**) Blood glucose during IPGTT (2 g/kg) in HFHS mice 8 h after IP injection of agonist (0.24 nmol/kg), *n*=8 per group, two-way repeated-measures ANOVA with Tukey's test, significance shown for exendin-phe1 *vs.* exendin-4. (**f**) AUC calculated from (d), relative to baseline glucose at t=0, one-way ANOVA with Tukey's test. (**g**) Cumulative food intake in fasted HFHS mice after IP injection of agonist (0.24 nmol/kg), *n*=8 per group. (**h**) As for (c) but 0.24 nmol/kg, *n*=10-11 per treatment. (**i**) Quantification of pica behavior from (i), Mann-Whitney test comparing exendin-4 *vs.* exendin-phe1. (**j**) As for (d) but agonist dose 0.24 nmol/kg, cherry Kool-Aid. (**k**) Blood glucose during IPGTT (2 g/kg) in lean, chow-fed mice performed 8 h after IP injection of indicated agonist (2.4 nmol/kg), *n*=4 per group. (**l**) Representative liver haematoxylin/eosin stained sections after 16 days continuous administration of agonist (0.24 nmol/kg/day) or vehicle, as used to calculate NAS score (Fig. 9g); scale bars, 10 µm. Data expressed as mean ± SEM. * p<0.05, ** p<0.01, *** p<0.001, by statistical test defined in the text.

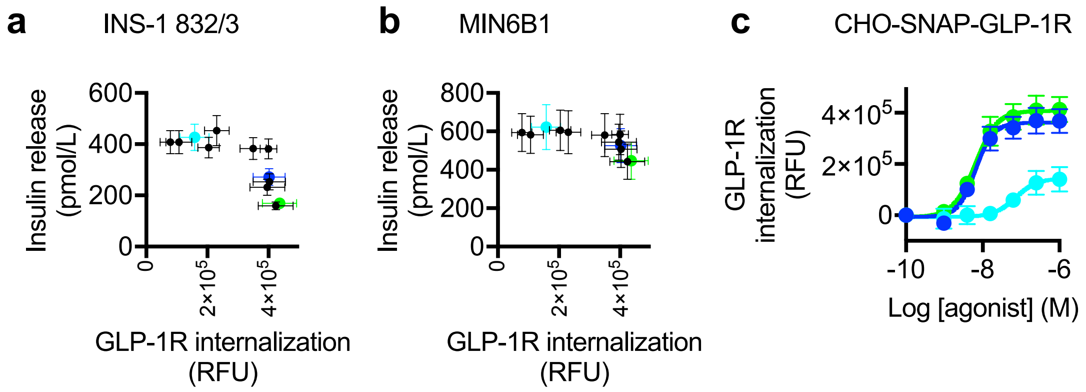

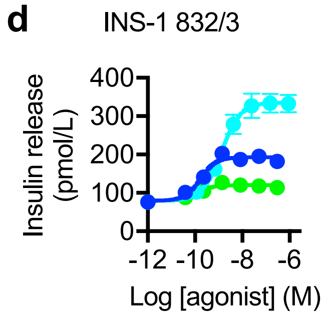

e FACS trafficking, MIN6B1-SNAP-GLP-1R

Ex-4	Exp1		Exp2		Exp3		Exp4		Exp5	
Time (min)	+MesNa	-MesNa	+MesNa	-MesNa	+MesNa	-MesNa	+MesNa	-MesNa	+MesNa	-MesNa
0	1358	4007	474	1677.3	431	1279.5	726.5	2656.5	823	2469
15	3481	3807	1740	1807	918	1034	2900	2910	2241	2489
30	3437	3872	1700	1877	997	1055	2713	2810	1887	2094
60	3541	3848	1750	1844	801	863	2285	2366	1830	2081

Ex-Phe1	Exp1		Exp2		Exp3	
Time (min)	+MesNa	-MesNa	+MesNa	-MesNa	+MesNa	-MesNa
0	1358	4007	705	2821	654	2885
15	1970	3640	1031	2224	993	2218
30	2695	3973	1204	2303	879	1630
60	2531	3519	1178	1954	1067	1684

Ex-Asp3	Exp1		Exp2		Exp3	
Time (min)	+MesNa	-MesNa	+MesNa	-MesNa	+MesNa	-MesNa
0	1358	4007	1187	3599	874	3615
15	3008	3467	3606	3984	3101	3664
30	3430	3914	3285	3358	2948	3307
60	3232	3743	3240	3285	2967	3143

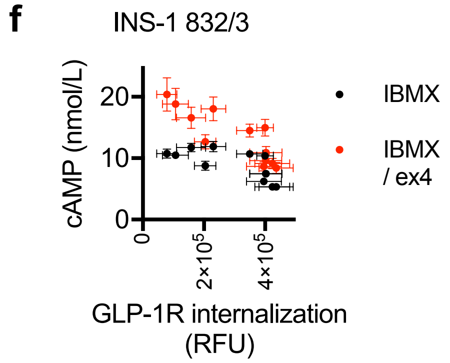

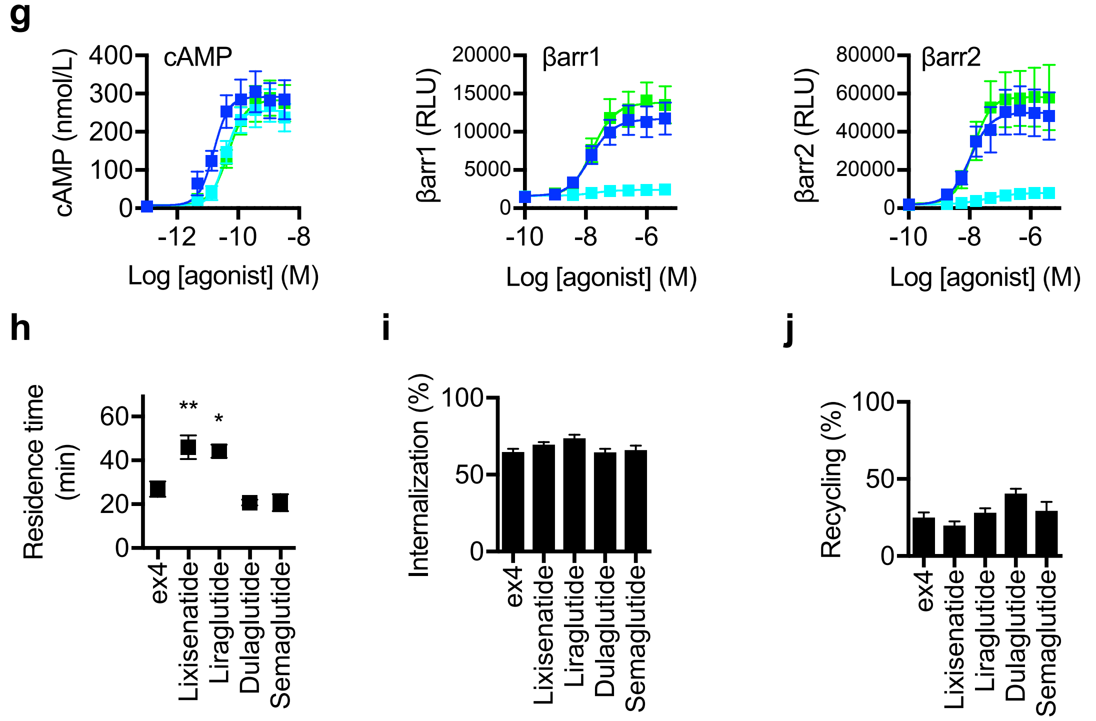

Supplementary Figure 10: Selected non-normalized data sets.

(**a**) Results from Fig. 1a, replotted to show absolute supernatant insulin concentration and loss of Lumi4-Tb-labelled cell surface SNAP-GLP-1R as relative fluorescence units (RFU). (**b**) As for (a), but referring to Fig. 1b. (**c**) Results from Fig. 1f, replotted to show loss of Lumi4-Tb-labelled cell surface SNAP-GLP-1R in RFU. (**d**) Results from Fig. 1g, replotted to show absolute supernatant insulin concentration. (**e**) Raw data from Fig. 2b, given here as median fluorescence units for each experiment. (**f**) Data from Fig. 3e, replotted to show absolute cAMP concentration against loss of Lumi4-Tb-labelled cell surface SNAP-GLP-1R in RFU. (**g**) Dose responses in PathHunter CHO-K1 cells replotted from Fig. 4c to show absolute cAMP concentration, and β-arrestin recruitment in relative luminescence units (RLU). (**h**) Agonist residence time, replotted from data in Fig. 6a to show absolute residence time in min. (**i**) GLP-1R internalization, as shown in Fig. 6e, replotted relative to vehicle treatment. (**j**) SNAP-GLP-1R recycling, as shown in Fig. 6f, replotted as % of internalized receptor undergoing recycling back to plasma membrane. Error bars indicate SEM.

Figure 2h

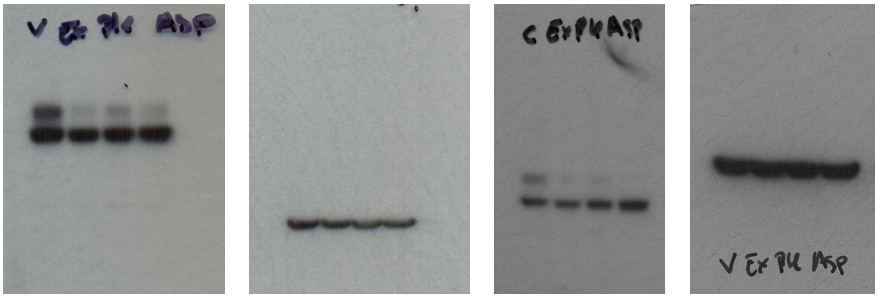

Supplementary Figure 5c

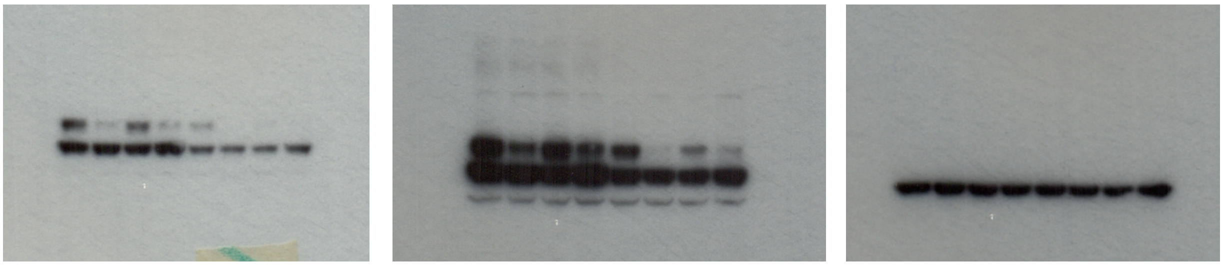

Supplementary Figure 7c, d, f

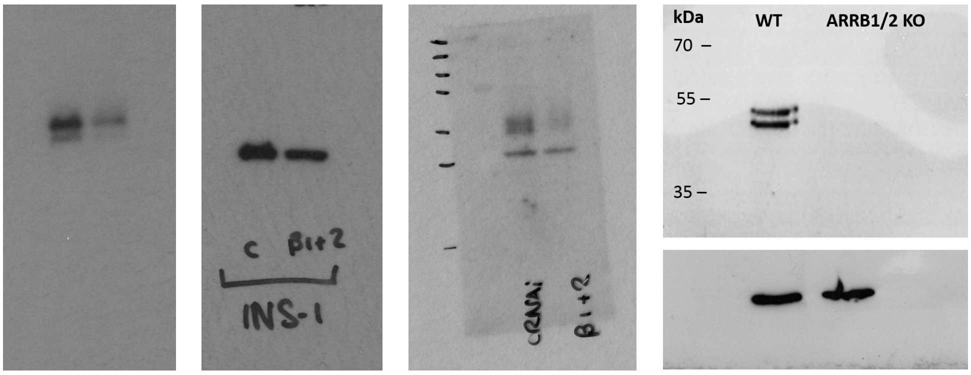

Supplementary Figure 11: Full scans of all blots presented in cropped form in figures in the primary and supplementary manuscript texts.

Figure 2e

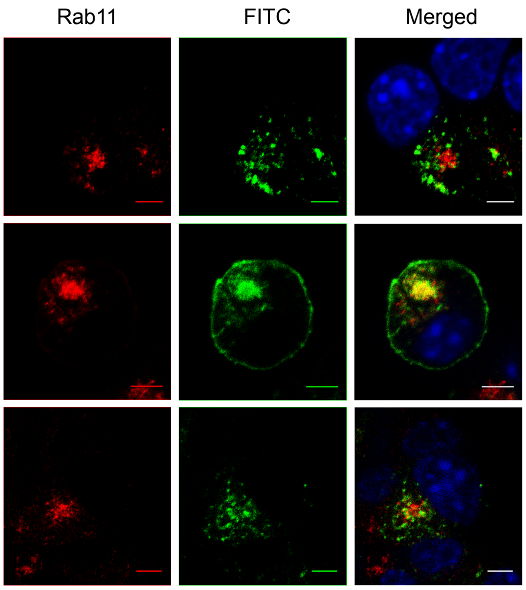

Figure 5e

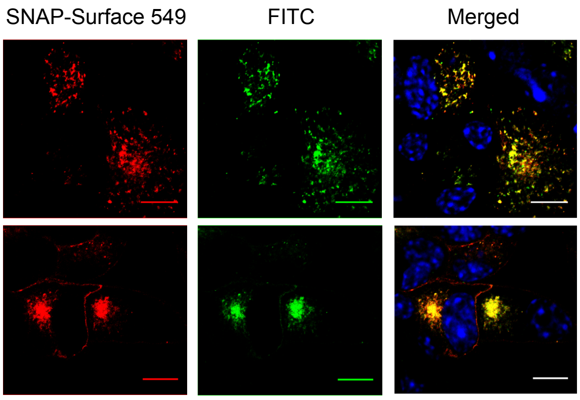

Figure 4e

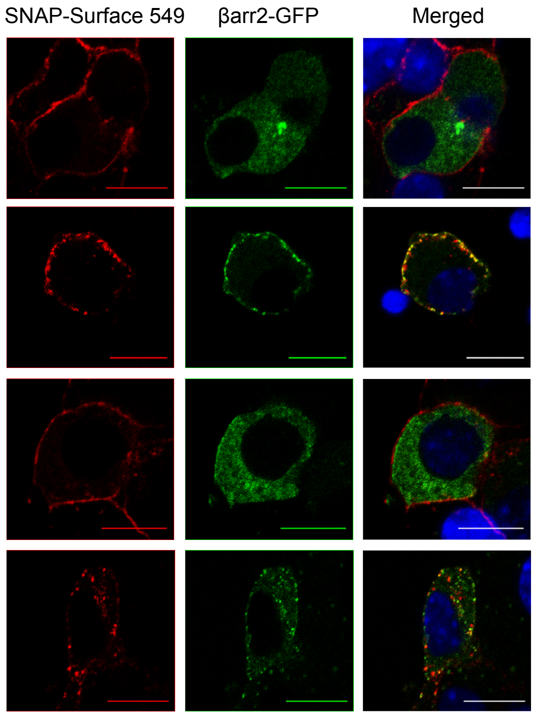

Figure 7a

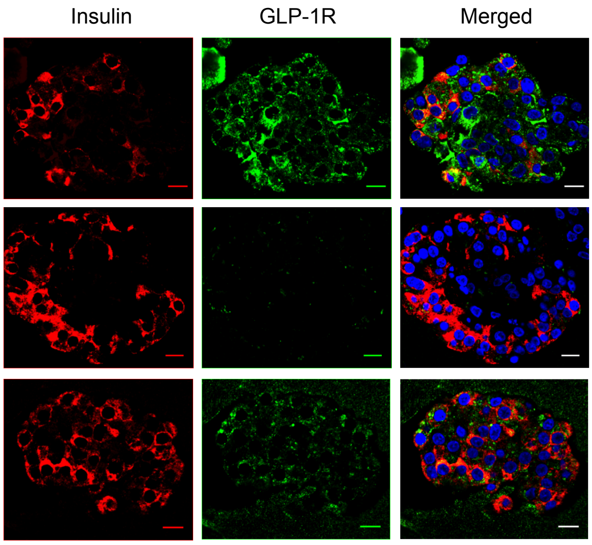

Supplementary Figure 12: Individual red and green channels from merged RGB images shown in the main figures.

Blue in merged, DAPI.

Target and description	Sequence(s)
Human *arrb1*, lentiviral shRNA	GAACTGCCCTTCACCCTAATG
Human *arrb2*, lentiviral shRNA	CTTCGTAGATCACCTGGACAA
Mouse *arrb1*, siRNA	UGGAUAAGGAGAUCUAUUA<br>GGAGAACCCAUCAGCGUUA<br>UCAUAGAGCUUGACACCAA<br>ACGGGAAGCUCAAGCAUGA
Mouse *arrb2*, siRNA	GGGCCUGUCUUUCCGCAAA<br>CUACUUGAAGGACCGGAAA<br>AUACCAACCUCAUCGAAUU<br>GUGCCAAAUCAAUAGAAGA
Rat *arrb1*, siRNA (Silencer Select s129662)	GAACUGCCCUUUACCUUAATT
Rat *arrb2*, siRNA (Silencer Select s129665)	GCUUAUCAUCAGAAAGGUATT

Supplementary Table 1: Target shRNA / siRNA sequences used in this study.

Date	Isolation centre	Age	Gender	BMI (kg/m^2)
09/02/2016	Geneva	37	Female	25.2
07/04/2016	Pisa	72	Male	27.4
13/04/2016	Edmonton	42	Male	33.3
20/04/2016	Edmonton	54	Male	28.7
19/05/2016	Pisa	61	Male	23.1
25/05/2016	Pisa	58	Male	26.1
27/05/2016	Edmonton (Macdonald)	55	male	23.6
01/06/2015	Edmonton (Macdonald)	28	Male	23.4
01/06/2016	Edmonton (Shapiro)	25	Male	20.8
01/06/2016	Oxford	63	Male	23
08/06/2016	Edmonton (MacDonald)	54	Female	23.4
15/06/2016	Edmonton (Shapiro)	36	Female	28.8
12/07/2016	Pisa	68	Male	21.5
14/07/2016	Milan	61	Male	27.8
22/09/2016	Pisa	75	Male	22.9
23/09/2016	Edmonton (MacDonald)	66	Female	33.1

Supplementary Table 2: List and characteristics of human islet donors included in this study, including date and location of isolation.

Target and description	Sequence(s)
Human *arrb1*	F: GCGGTGTGGACTATGAAGTCAA<br>R: ACAGAATTCCGCTTGTGGATCT
Human *arrb2*	F: GACCGTCAAGAAGATCAAAGTCTCT<br>R: GAGATACCTGGTCATCTTGTTCGA
Mouse *arrb1*	F: CCAGACAGTTCCTTATGTCAGACAA<br>R: TTCTCCGTGGTAATAGATCTCCTTATC
Mouse *arrb2*	F: ACCACACGCCACTTCCTCAT<br>R: CCCGTGGTAGTACAGCTCTTTGT
Rat *arrb1*, Taqman assay Rn01648673_m1	Amplicon context sequence:<br>GAAGCTGGGCGTTGAGATCCCGCCAAACCTTCCGTGCTCAGTCATTGAGATCCCGCCAAACCTTCCGTGCTCAGTCA
Rat *arrb2*, Taqman assay Rn01456874_g1	Amplicon context sequence:<br>CCACTTCCTCATGTCTGACCGGAGGTCCCTGCACCTAGAGGCTTCCCTGGACAAAGAGCTGTACTACCATGGGGAACCCCTCAATGTCAACGTCCACGTCACCAACAATTCTGCCAAGACCGTCAA

Supplementary Table 3: qRT-PCR primer sequences used in this study.
